# Book Review: Networks of the Brain

**DOI:** 10.3389/fpsyg.2017.01299

**Published:** 2017-08-02

**Authors:** Fozia Anwar, Afifa Yousafzai, Muaz A. Niazi

**Affiliations:** COSMOSE Research Group, Health Informatics, COMSATS Institute of Information Technology Islamabad, Pakistan

**Keywords:** neuronal networks, complex adaptive systems, complex networks, social network analysis, complex systems

## Introduction

Natural systems consisting of living systems as well as some artificial systems (Altamimi and Ramadan, 2016; Batool and Niazi, 2017) can be classified as Complex Adaptive Systems (CAS) (Holland, 2012). These systems have complexity in terms of having nonlinear interactions between numerous agents—interactions which cannot be easily summarized. CAS also have some rather peculiar properties—they adapt such that individual agents do not matter—an example being of cells which can continually get replaced with no discernable loss of overall emergent functionality. Examples of CAS include humans or animals. Such systems simply cannot be understood by dissection or only from the perspective of being made up of a wide variety of cells or elements/compounds for that matter.

CAS are all around us and even inside us. Human beings consist of numerous hierarchical and intertwined CAS both individually as well as collectively. CAS are so close and abundant on this planet that it is actually very difficult think of anything with no link to a CAS—hence we cannot see the forest for the trees. The only way to make sense of CAS seems to be to develop various types of models or simplified abstractions of complex real-world systems. Modeling takes out any unnecessary details—example being of maps (Miller and Page, 2007). Maps provide information about the real-world and yet do not cover every possible detail. Likewise, agents and networks can be used to model the real world or concepts inspired (Zedadra et al., 2017) by the real world.

Like maps, there are models which are more appropriate to modeling CAS. These include agent-based, complex network based (Mrvar and Batagelj, 2016), and multiscale models. The book which is being reviewed here applies the complex network approach to modeling the brain.

## Review

The goal of this book is to exemplify the importance of and usage of network science in neuroanatomy. The book gives a holistic approach to the complex network perspective including its historical roots.

The book has four portions. The first one has three chapters covering the basic details of the two domains. The second portion, consisting of chapters 4–7, focuses on the anatomical networks of cells and regions. Whereas the third portion of the book (chapters 8–11), is dedicated to network dynamics. The final portion has three chapters covering different aspects of network complexity.

Chapter 1 eloquently explains the similarities between brain networks architecture and architecture of complex systems. It also motivates some key neuroscience questions which could be effectively addressed using network models. Chapter 2 introduces the basics both in terms of terminologies as well as methodologies using in network science. It also includes as perceptive survey of the quantitative tools and concepts which could potentially be useful for brain-related studies. Chapter 3 describes some fundamental techniques and approaches used to mine and extract brain networks from neurological data.

The second portion starts with Chapter 4 offering a network perspective to the functional anatomy of the brain including its historical provenance. An emerging set of ideas is also outlined regarding functional and structural specialization. Chapter 5 outlines modern neuroanatomical techniques useful for mining brain networks. Chapter 6 presents some so well-known key architectural principles of anatomical networks. Chapter 7 examines the functional meaning and evolutionary origins. The chapter examines the so-called “wiring minimization” hypothesis. This includes questions such as, to what extent has brain connectivity been shaped by spatial and metabolic constraints? Also, the optimal economy of the elements and connections of brain networks (in a spatial or metabolic sense) is examined besides some other questions.

The third portion of the book begins with Chapter 8 focusing on functional networks generated by spontaneous activity in neural systems. Chapter 9 attempts to draw links between brain networks and cognition. Chapter 10 outlines existing knowledge about brain network disruptions in neurological and psychiatric diseases. Chapter 11 focuses on the growth, development, and aging of brain networks. Chapter 12 makes the case for diverse and flexible neural dynamics as a prerequisite for efficient computation. Chapter 13 traces the origin of complex dynamic patterns for structural patterns of network connectivity. Finally, chapter 14 examines the role of the body in shaping the functioning of brain networks.

## Critical comments

The book is generally well-written but is probably not suitable as a first book in Network Science. It is a thoroughly technical book. Easy and nonmathematical approach of this book towards the subject still make this book a good read. References used in this book are also up-to-date. An appropriate first book on networks could be the text by Newman (2010).

## Conclusions

Olaf Sporns has presented a readable treatise for readers interested in complex systems as well as those interested in areas ranging from quantitative psychology to neurosciences. The idea of modeling complex neuronal structures as networks is quite new. While there are some previously published books in this domain, there was a need for a comprehensive text on this topic. Although the book does not introduce complexity-related details it is still a very interesting read and highly recommended as a ready reference.

## Author contributions

MN conceived the idea of the paper. MN, FA, and AY all wrote and edited the paper. All authors read and approved the final manuscript.

### Conflict of interest statement

The authors declare that the research was conducted in the absence of any commercial or financial relationships that could be construed as a potential conflict of interest.
